# A generalized machine learning model for long-term coral reef monitoring in the Red Sea

**DOI:** 10.1016/j.heliyon.2024.e38249

**Published:** 2024-09-21

**Authors:** Justin J. Gapper, Surendra Maharjan, Wenzhao Li, Erik Linstead, Surya P. Tiwari, Mohamed A. Qurban, Hesham El-Askary

**Affiliations:** a Earth Systems Science and Data Solutions Lab, Chapman University, Orange, CA, 92866, USA; b Schmid College of Science and Technology, Chapman University, Orange, CA, 92866, USA; c Fowler School of Engineering, Chapman University, Orange, CA, 92866, USA; d Center for Environment and Water, The Research Institute, King Fahd University of Petroleum and Minerals (KFUPM), Dhahran, 31261, Saudi Arabia; e National Center for Wildlife, Saudi Arabia; f Department of Environmental Sciences, Faculty of Science, Alexandria University, Moharem Bek, Alexandria, 21522, Egypt

**Keywords:** Spatiotemporal coral change analysis, Machine learning, SVM, Classification, Landsat

## Abstract

Coral reefs, despite covering less than 0.2 % of the ocean floor, harbor approximately 35 % of all known marine species, making their conservation critical. However, coral bleaching, exacerbated by climate change and phenomena such as El Niño, poses a significant threat to these ecosystems. This study focuses on the Red Sea, proposing a generalized machine learning approach to detect and monitor changes in coral reef cover over an 18-year period (2000–2018). Using Landsat 7 and 8 data, a Support Vector Machine (SVM) classifier was trained on depth-invariant indices (DII) derived from the Gulf of Aqaba and validated against ground truth data from Umluj. The classifier was then applied to Al Wajh, demonstrating its robustness across different sites and times. Results indicated a significant decline in coral cover: 11.4 % in the Gulf of Aqaba, 3.4 % in Umluj, and 13.6 % in Al Wajh. This study highlights the importance of continuous monitoring using generalized classifiers to mitigate the impacts of environmental changes on coral reefs.

## Highlights

• The classifier achieved 78.22 % accuracy in Aqaba and 72.73 % in Umluj.
• Coral reefs loss ranging from 3.4 % to 13.6 %.
• Landsat data from 2000 to 2018 revealed significant habitat loss.

## Graphical abstract

**Figure:**
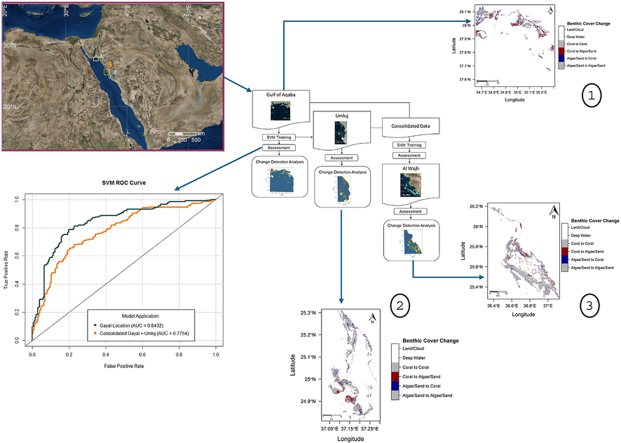

## 1 Introduction

It is noteworthy that coral reef habitats make up less than 0.2 % of the ocean cover yet contain a high value of ∼35 % of all known marine species [1]. Global bleaching events and their effect on coral reefs are well-documented [[2], [3], [4], [5], [6], [7], [8], [9], [10]].The main driver of coral reef degradation globally is coral bleaching [6]. Some researchers have now projected total loss of this critical environment [11,12]. The United States National Oceanic and Atmospheric Administration (NOAA) officially declared the third global coral bleaching event in October 2015. This event was significant due to record ocean temperatures, which caused widespread coral bleaching across Hawaii and other regions. The event was driven by climate change and exacerbated by the El Niño phenomenon, which led to prolonged high ocean temperatures affecting coral reefs worldwide [13]. Therefore, it is urgent to monitor coral reefs to mitigate further degradation and protect these vital ecosystems.

Remote sensing is a valuable tool for monitoring coral reefs due to its ability to provide extensive and frequent observations over large and remote areas [[14], [15], [16], [17], [18]]. NASA's Landsat sensors are known to be used for change detection analysis and has been used for coral reefs change detection studies [[19], [20], [21]]. Many studies were performed to isolate an individual reef or location for a specific change detection analysis [19,22,23]. These in-situ style analyses all share a similar framework for analysis. First, a primary state image and a subsequent state image of the reef are identified. Then, the subsequent state image is used to train a simple classifier then applied to the primary state image. Finally, change analysis is performed by a per pixel comparison between the two results [[24], [25], [26], [27]]. This framework is well established and effective in ascertaining change in specific, isolated, and individual locations (i.e. in-situ locations) [28]. However, these frameworks are specific and inflexible due to the high cost of obtaining ground truth data for training classifiers [29]. This limitation results in a finite and constrained approach, as each study requires a set of ground truth observations for classifier training [30]. Consequently, change detection analysis is impractical without ground observations for the target location. While unsupervised learning methods can address this issue, they are generally less accurate than supervised methods when applied to large datasets [31].

Therefore, expanding in-situ approaches is a critical next step in advancing coral reef change detection research. The in-situ approach can extend beyond the scope of a single, isolated reef if a clear extrapolation process is developed to generalize results longitudinally [32,33]. This study outlines a method for utilizing combined information from multiple locations to train and develop a robust machine learning classifier with general applicability. Since long-term change detection of coral reef habitats using satellite observations has been limited assessed in the Red Sea areas, with little research reported over an 18-year period [34]. The increasing frequency and severity of coral bleaching events highlight the importance of this research in recognizing and evaluating the impacts on Red Sea coral reef habitats, including those due to climate change [35,36].

## 2 Materials and methods

### 2.1 Study area

The Red Sea is a long and narrow basin, lying between Africa and Asia. Saudi Arabia and Yemen are located to the east of the Red Sea in the Arabian Peninsula, while Egypt, Sudan, and Eritrea are located to the west in Africa. The Red Sea is a semi-enclosed, elongated basin, approximately 2000 km long, up to 355 km wide, and a mean depth of 524 m [37]. The Red Sea water mass exchanges its water with the Arabian Sea, the Indian Ocean via the Gulf of Aden [37,38]. The connection of the Red Sea to the Indian Ocean is in the south through the Bab-al-Mandab strait and the Gulf of Aden [37]. In the north, there is the Sinai Peninsula, the Gulf of Aqaba, and the Gulf of Suez connecting to the Suez Canal. The Gulf of Suez is a shallow basin with maximum depths of <100 m that connects the Red Sea to the Mediterranean Sea while the Gulf of Aqaba has maximum depths of >1800 m and is connected to the Red Sea through a 260 m deep sill in the Strait of Tiran [39].

Because of its arid setting and a very peculiar and diverse environment, low precipitation, and high evaporation occur during the summer season [40]. The physical forcing reduces the effect of salinity caused by the evaporation in the north and relatively hot water in the southern Red Sea. The rainfall over the Red Sea and its coasts are extremely low, averaging 0.06 m (2.36 inch) per year [41]. The Red Sea undergoes strong atmospheric forcing through wind stress, heat, and precipitation fluxes.

This investigation initially assessed a beachfront locale close to the city of Gulf of Aqaba. Counting Sanafir Island, the space of interest (AOI) ranges from roughly 35°20′E to 34°35′E and 27°30′N to 28°50′N. Second, a gathering of reefs off the shoreline of Umluj crossing from roughly 36°55′E to 37°20′E and 24°35′N to 25°20′N were considered. Because of the encompassing scene, little water from waterways enter the Red Sea. This result in a little variety in water saltiness, temperature, and quality [37].

The coral reefs of the Red Sea are characterized by high biodiversity and endemism, with over 300 species of hard corals and a wide array of associated fauna, including fish, invertebrates, and algae. These reefs are typically found in oligotrophic waters, with low nutrient levels and high water clarity, fostering the growth of various coral species. The reefs at the study sites exhibit a mix of fringing, barrier, and patch reefs, providing a diverse range of habitats.

Gulf of Aqaba: Located at the northernmost tip of the Red Sea, the Gulf of Aqaba is known for its unique coral reef ecosystems that thrive in high salinity and relatively cooler waters. The reefs here exhibit high coral cover and diversity, with a notable presence of endemic species. The Gulf of Aqaba is also recognized for its resilience to thermal stress, making it an important site for studying coral adaptation to climate change [42,43].

Umluj: Situated along the central Red Sea coast, Umluj is often called the "Maldives of Saudi Arabia" due to its pristine beaches and rich marine biodiversity. The reefs in Umluj are predominantly fringing reefs with extensive coral gardens and vibrant fish communities. This area is less impacted by human activities, providing an excellent site for studying relatively undisturbed coral ecosystems.

Located in the northern Red Sea, Al Wajh features a large and shallow barrier reef system that supports a unique fish community. The 1500 km^2^ lagoon experiences greater temperature and salinity fluctuations and higher turbidity than most other Red Sea reefs. These conditions influence coral community structure and introduce physiological challenges to its resident organisms, resulting in a distinctive reef-associated fish community [44].

### 2.2 Preprocessing steps and data used

In this work two remote sensing platforms with 30-m spatial resolution and three visible channels were used, namely the Landsat 7 Enhanced Thematic Mapper (ETM) and Landsat 8 Operational Land Imager (OLI) in 2000 and 2018, respectively. Landsat 8 bands 2, 3 and 4 ranging (0.450–0.515 μm), (0.525–0.600 μm), and (0.630–0.680 μm), respectively, were utilized for this investigation. This is due to their water segment infiltrating properties, while the coastal aerosol band, band 1, was not used because the Landsat 7 mission offers no comparable data. The initial condition of each site was analyzed using Landsat 7 band 2 (0.45–0.51 μm), band 3 (0.53–0.59 μm), and band 4 (0.64–0.67 μm). Both Landsat 8 and Landsat 7 maintain a revisit cycle of 16-days. Landsat 8 scenes from 2018 were selected to minimize obscurities including cloud cover from within the AOI of the scene. Similarly, Landsat 7 images from 2000 were selected after taking into account the obscurities and cloud cover. Table S1 displays the site name, scene capture dates, AOI dimensions, and number of ground truth observations for each of the sites.

Ground truth observations were obtained through visual census (VC) conducted by a team from King Fahd University of Petroleum and Minerals (KFUPM) in Saudi Arabia. The survey covered three regions, each comprising multiple sites. At each station, a diver deployed a 30 m long transect along the reef slopes and adjacent areas. A digital camera in a waterproof housing was mounted on a PVC frame. The frame was positioned randomly on both sides of the transect, and photographs were taken at regular intervals. Each photograph was analyzed using the Coral Point Count program, which applied 100 random points to compute the percent cover of the following categories: Sand, Rock, Mud, Rubble, Coral, Seagrass, and Seaweed. The resulting survey data was subsequently processed for model training and validation.

### 2.3 Preprocessing

This work started by acquiring tier 1 data products from 2000 to 2018 over the regions of interest and performing a radiometric characterization and geometric correction to obtain level-1 Precision and Terrain corrected data (L1TP). The data were determined to have well-characterized radiometry within image-to-image tolerances of ≤12-m radial root mean square error (RMSE) (Landsat project science office, June and October 2018). A mask was applied using the Landsat Quality Band (BQA) to remove any obscured pixels including clouds [45,46]. Using the Landsat near infrared (NIR) bands, a water mask was also developed, by identifying areas of full wavelength absorption, because in that spectral range (0.851 and 0.0879 μm), light does not penetrate water [47]. On the other hand, the dark-pixel subtraction approach was used to perform the atmospheric and sun glint corrections [28,[48], [49], [50], [51], [52]]. The method of Lyzenga was applied for water column correction yielding the per-pixel depth invariant index (DII) [15,[53], [54], [55]].This method utilized the ratio of attenuation coefficients between each pair of bands negating the need to calculate estimates of k for each band directly [15,54]. To calculate the value of DII the following equation is used.

$${DII}_{ij}=\ln\left(L_{i}-L_{si}\right)-\left[\frac{k_{i}}{k_{j}}∙\ln\left(L_{j}-L_{sj}\right)\right]$$

Where, $L_{i}$ and $L_{si}$ represent the radiance at sensor and mean deep-water radiance in band *i*, respectively. Moreover, $a_{i}$ is a constant for band *i* that accounts for atmospheric effects and water surface reflection, where $r_{i}$ corresponds to the bottom reflectance and z for depth. Finally, f is a geometric factor accounting for path length through water (set to two for a two-flow model) and $k_{i}$ represents the coefficient of attenuation of band *i* (to account for scattering due to turbidity and various interferences suspended in water) [28,55].

The ratio of attenuation between each pair of bands can be determined as:

$$\frac{k_{i}}{k_{j}}=a+\sqrt{\left(a^{2}+1\right)}$$

Where

$$a=\frac{\sigma_{ii}-\sigma_{jj}}{2\sigma_{ij}}$$

and $\sigma_{ii}$ is the variance of band i and $\sigma_{ij}$ is the covariance between bands i and j. Consequently, using these equations, we can determine DII for some random scene with no need to reference other external information. Our proposed machine learning classifier benefited from the DII values, as inputs, resulting from the preprocessing steps.

### 2.4 Generalized machine learning classifier for coral reefs

Our methodology started by training the classifier using the DII values from the preprocessed images of the Gulf of Aqaba only. This delivered a standard for assessment of how well the calculation would sum up to extra locales. A separated irregular inspecting of the perception and DII information was applied to adapt to class unevenness. The DII values derived from the Landsat 8 image of the Gulf of Aqaba were applied as inputs to the SVM algorithm and validated against the ground truth observations. We then performed hyperactive parameter tuning using cross-validation to identify the optimal gamma values and the cost associated with model accuracy and robustness. In our model, a radial basis kernel function outperformed other kernels while we were careful enough to avoid model overfitting to enable robust generalization. Additional input features such as entropy were attempted that contributed to increasing the classification accuracy, yet these additional features did not show good generalization based on the cross-validation performance. We used six different measures to evaluate the tuned SVM model namely, specificity, precision (1-user error), recall (producer error), accuracy (percentage of ground truth observations correctly classified), kappa coefficient, and F-measure.

Accuracy is a simple measure of the percentage of ground truth observations correctly classified. Six different validation metrics were used to evaluate the generalization of the trained SVM model: precision, recall, specificity, accuracy, F-measure, and kappa coefficient. Precision and recall are statistical values to measure performance against type I and type II errors, respectively. Type I errors represent false positive predictions while type II errors represent false negative predictions. Recall measures the completeness of the result while precision measures the model's exactness. Precision is equal to (1−usererror) and recall is equivalent to (producererror). Specificity is also known as the true negative rate. This is an additional evaluation criterion which measures the number of true negatives that are correctly identified. The harmonic mean of precision and recall is the F-measure. This model performance measurement is effective in evaluating the balance between precision and recall and the overall accuracy of the model. The kappa coefficient is a measure of the ratio of correct classification with respect to baseline agreement. This metric has come under scrutiny particularly when there is significant class imbalance, as is the case with most coral cover detection analysis. Still, the metric can be informative in coordination with other metrics leveraged for this study.

After the SVM model was evaluated, it was applied to the DII values from the entire Landsat 7 and Landsat 8 scenes. The posterior probability of each pixel was used to develop a map of coral cover. A threshold was applied to these probabilities to yield a predicted class, which also was mapped. Change detection analysis for coral cover was conducted via a per-pixel comparison of the initial class and the final class for the Gulf of Aqaba site.

**Fig. 1:**
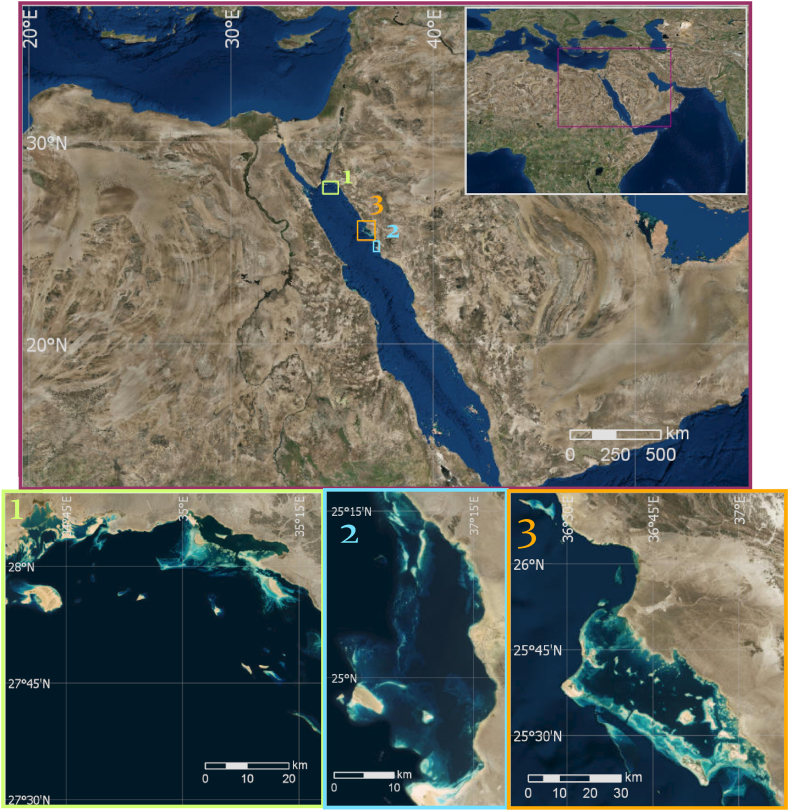
Base map of the areas under investigation: 1) Gulf of Aqaba, 2) Umluj and 3) Al Wajh.

The algorithm trained using the ground truth observations and DII values from the Gulf of Aqaba site was then applied to predict the ground truth observations of the Umluj location. This process leverages the well-known data science practice of train and test split in order to develop a true evaluation of model robustness. Specifically, this method identifies how well a machine learning model developed using data from one reef can predict the coral cover in a separate, unobserved reef. Performance against the Umluj site was evaluated using the same assessment criteria as was applied to the Gulf of Aqaba site (specificity, precision, recall, accuracy, kappa coefficient, and F-measure). Once evaluated, a map of the per-pixel posterior probability for coral class was created for the Umluj location. The per-pixel predicted class was developed by applying a threshold and the associated map of each pixel class. Change analysis was developed through a pixel-by-pixel comparison between the initial state classification and the final state classifications. A diagram of this procedure is outlined in Fig. S1 (see Fig. 1).

**Fig. 2:**
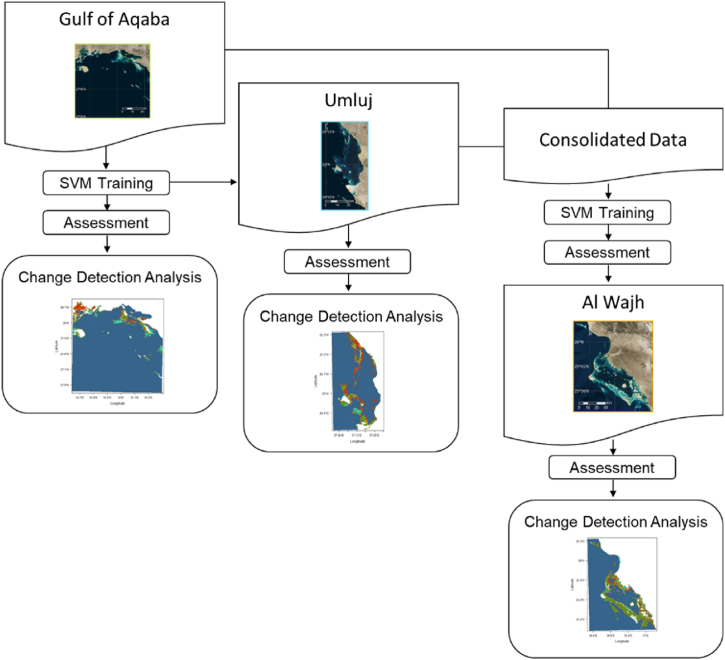
Generalized SVM Classifier training and change analysis process flow.

A vigorous AI classifier was created following the model assessment applied to the Gulf of Aqaba area and Umluj area. This classifier was prepared utilizing the joined ground truth perceptions of both Gulf of Aqaba and Umluj. The dependent variable for this classifier were the consolidated ground truth observations and the independent variables were the associated DII pixel values from both Gulf of Aqaba and Umluj. Change analysis of the Al Wajh location was performed by comparing the per-pixel predicted class using the robust classifier. A diagram of this procedure is outlined in Fig. 2.

Generalization of new information from additional sites is accomplished using the consolidated information from both Gulf of Aqaba and Umluj [20]. This outcome is because of the reduction in site-specific bias that results from training the model on information that is more representative of the larger ecology of the area. Rather than memorize the hyper specific geomorphology, strata, turbidity, and fauna of an isolated, individual reef, the robust classifier targets modeling a much larger extent of reef habitats. The outcome is a calculation that is more representative of the region as opposed to the particular area-based bias that in-situ investigations depend upon. Preparing the SVM utilizing information that is representative of the region as opposed to a particular reef empowers a bigger degree investigation of coral reefs than previously possible [21]. Analysis was performed using the open-source R programming language and environment for statistical computing [56]. The main aim of our study was to evaluate the effectiveness of the SVM algorithm in classifying coral cover using historical data. We focused our analysis on data from 2000 to 2018 to ensure methodological rigor and validate the algorithm, avoiding the complexities and potential inconsistencies of newer data. Significant changes in coral reefs occurred during this period, as documented by various studies. For instance Ref. [57], reported recurrent mass bleaching events globally between 1998 and 2016 due to global warming, highlighting severe impacts on coral cover. Similarly [58], discussed the effects of climate change and ocean acidification on coral reef ecosystems up to 2017, indicating substantial changes. Additionally [59], found a significant decline in coral cover on the Great Barrier Reef from 1985 to 2012. These studies underscore the substantial changes in coral cover before 2018, supporting our focus on this period for algorithm validation.

## 3 Results

### 3.1 Classification accuracy by site

Table S2 includes the outcomes from each measure as applied to the Gulf of Aqaba location data and Umluj location data. The Gulf of Aqaba location included 1085 ground truth observation points identified as coral cover. Class imbalance was reduced through stratified random sampling of the observations in order to ensure a more robust classification of pixel coral cover. 404 observations were included in the sampled data 78.22 % of which were correctly classified by the tuned SVM classifier. Precision of 0.7664, recall of 0.8119, and an F-measure of 0.7885 was obtained by the classifier. Specificity measured 0.7525 and the kappa coefficient yielded by the model as applied to the Gulf of Aqaba location was 0.5644.

**Fig. 3:**
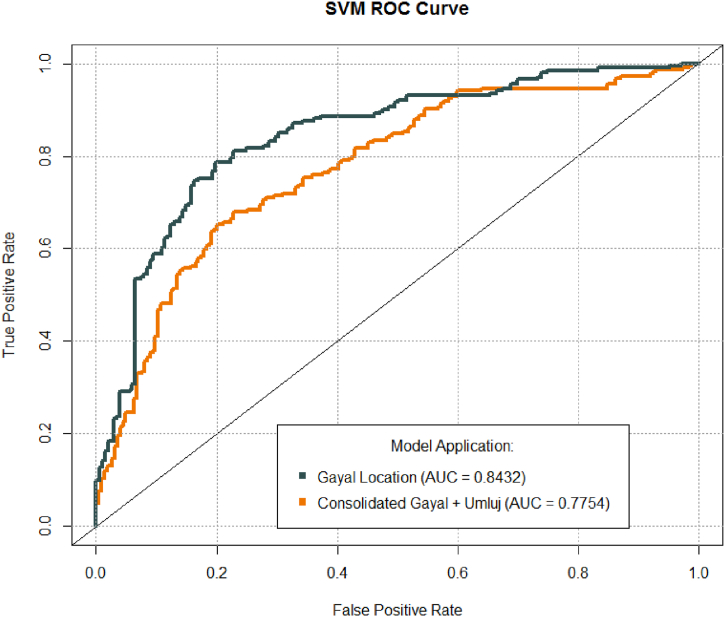
Performance evaluation of the robust, combined classifier using Receiver Operating Characteristic (ROC) Curve and Area Under Curve for the Gulf of Aqaba and consolidated model.

The algorithm trained using the Gulf of Aqaba data was applied to the truth observations from the Umluj data and yielded an accuracy of 72.73 %. Loss of site-specific bias that in-situ analysis depend on such as strata, geomorphology, turbidity, and local fauna resulted in a decrease in accuracy. This decrease was expected due to the challenging research objective of training a model in one location and extrapolating that model to an entirely different location. Hence, the main objective of this exploration was to deliver an AI classifier that could sum up past the extent of an in-situ examination which is hyper restricted in degree. As a result, given the challenge of generalizing to entirely new locations, this decrease in accuracy is acceptable. In assessment of the Umluj perceptions, a defined arbitrary examining was utilized to adapt to class unevenness too. 44 ground truth perceptions were chosen and the model tuned on the Gulf of Aqaba information was applied. This resulted in a precision and recall of 0.7500 and 0.6818, respectively and the model yielded a specificity of 0.7727. The F-measure of the Umluj deployment was 0.7143 demonstrating a high level of balance between precision and recall. The model yielded a kappa coefficient of 0.4545. A summary of these figures is included in Table S2. Table S3 details the confusion matrices for the deployments at each site in addition to results produced by the robust model trained from the consolidated datasets. The robust classifier receiver operating characteristic (ROC) curve as well as the ROC curve is included in Fig. 3. The ROC curve performance resulting from the Gulf of Aqaba is also included for comparison. The ROC is a common tool for visualizing the tradeoff and overall performance of a classifier. It plots to recall or the true positive rate as a function of 1−Specificity or the false positive rate. The area under the ROC curve (AUC) is another common diagnostic tool for measuring model performance. The combined classifier yielded an AUC of 0.7754.

### 3.2 Consolidated model robust to site-specific bias

A powerful support vector machine model was developed using merged data from both the Gulf of Aqaba and Umluj sites. For this model, the features included a randomly selected sample of per-pixel DII values from both regions. The diversity of inputs resulting from combining data from multiple locations enabled the model to generalize more effectively to data from new areas. However, the generalization properties of the classifier trained under this method resulted in a minor decrease in accuracy. This reduction in accuracy occurs because the machine learning algorithm no longer benefits from site-specific information and biases, such as local geomorphology, water turbidity, and marine fauna. In-situ analyses leverage these site-specific biases to develop models that are highly accurate when applied to the individual reef containing the information from which they were trained. However, when generalizing these models beyond a single location, these biases represent overly specific training, analogous to the common data science principle of overfitting. Consequently, these classifiers perform well when applied to data from the specific location they were developed for but cannot generalize to new data beyond the limited scope of the in-situ extent.

Since the robust classifier is developed using inputs from multiple sites, its data sources are more representative of the broader benthic environments rather than a single reef within the region. Developing the model using inputs more representative of a larger extent allows it to evaluate reefs throughout the region more effectively, albeit at the cost of accuracy for any single reef within the area. Of the 448 ground truth pixels selected from the delineated random sampling of data from both the Gulf of Aqaba and Umluj areas, 318 were accurately classified, resulting in an accuracy of 70.98 %. Precision and recall were measured at 0.6992 and 0.7366, respectively, with a specificity of 0.6830. The F-measure was calculated at 0.7174, and the kappa coefficient was 0.4196. Table S3 presents the results of this evaluation. The corresponding confusion matrix is included in Table S3. The consolidated model's ROC is shown in Fig. 3, along with the model's performance based on the Gulf of Aqaba location data for comparison. The consolidated model obtained an AUC of 0.7754. The curves in this ROC chart represent the tradeoff in model accuracy as the classification threshold varies. The 45° line represents performance using random assignment of class to observations. Improved model performance can be observed as the curves deviate from this line.

### 3.3 Change detection analysis

#### 3.3.1 Gulf of Aqaba location

**Fig. 4:**
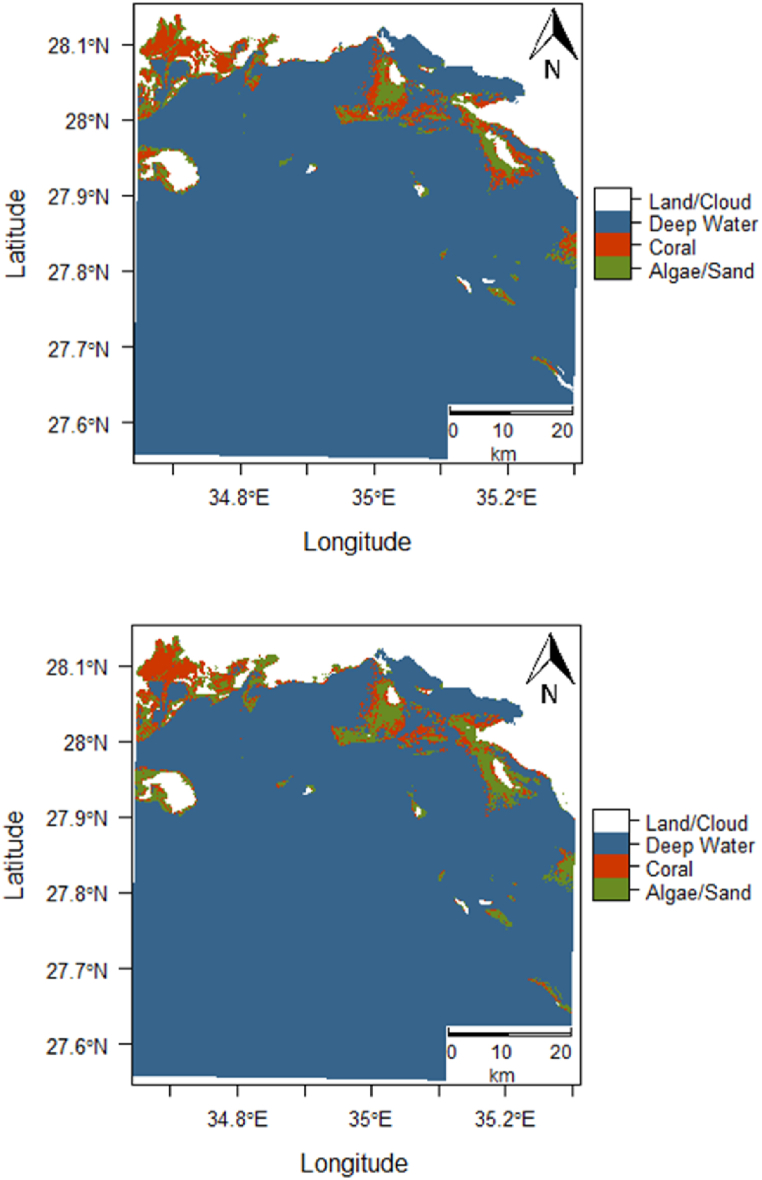
Predicted class membership map for the Gulf of Aqaba AOI (top, 2000, and bottom, 2018).

The initial Landsat 7 data for the Gulf of Aqaba, obtained on March 18, 2000, was analyzed against Landsat 8 data captured on November 7, 2018. The initial image contained 147,014 pixels identified as coral by the SVM classifier. Of these, 135,288 pixels were classified as sand, algae, or other benthic cover types, corresponding to 132.31 km^2^ of coral as can be seen in Fig. 4. In 2018, the final image showed 130,225 coral pixels and 152,077 non-coral pixels, equating to 117.20 km^2^ of coral, representing an 11.4 % reduction over the 18-year period.

**Fig. 5:**
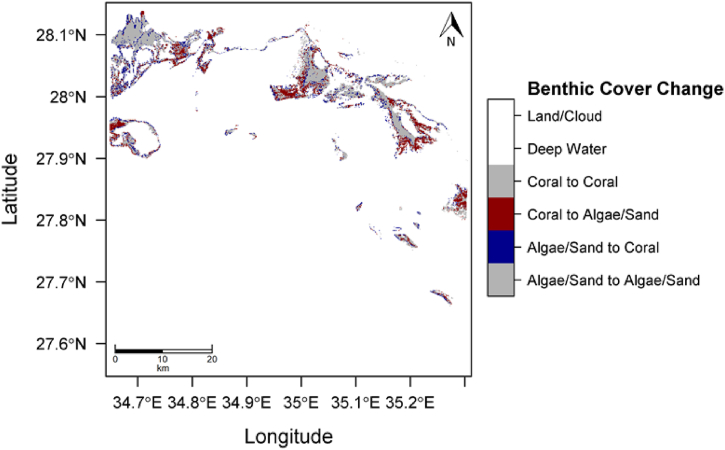
Change detection analysis between 2000 and 2018 of the Gulf of Aqaba AOI.

From this analysis, we found that in the initial state image, coral covered 52.1 % of the shallow benthic area, whereas in 2018, this coverage had decreased to 46.1 %. Fig. S2 presents the posterior probability maps for the initial 2000 image (top) and the final 2018 image (bottom). Fig. 4 shows the predicted classification maps based on the trained algorithm for 2000 (top) and 2018 (bottom A per-pixel difference over the 18-year period is displayed in Fig. 5.

#### 3.3.2 Umluj location

A lesser decline in coral cover was observed within the Umluj location compared to other study areas. The initial image from this location, captured on February 17, 2000, using the Landsat 7 platform, was compared to a Landsat 8 image taken on December 11, 2018. The baseline image included 113,284 pixels (101.96 km^2^) classified as coral by the SVM model. The final image showed 109,443 pixels (98.50 km^2^) classified as coral, indicating a 3.4 % reduction in coral cover over the 18-year period.

**Fig. 6:**
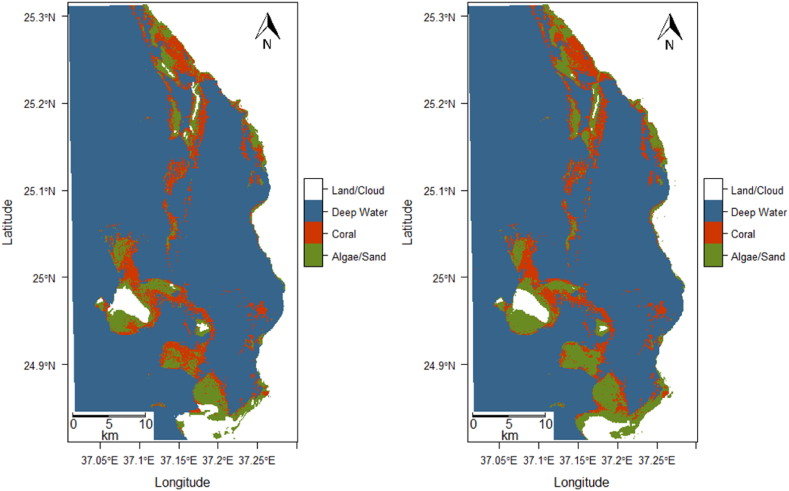
Predicted class membership map for the Umluj AOI (left, 2000 and right, 2018).

**Fig. 7:**
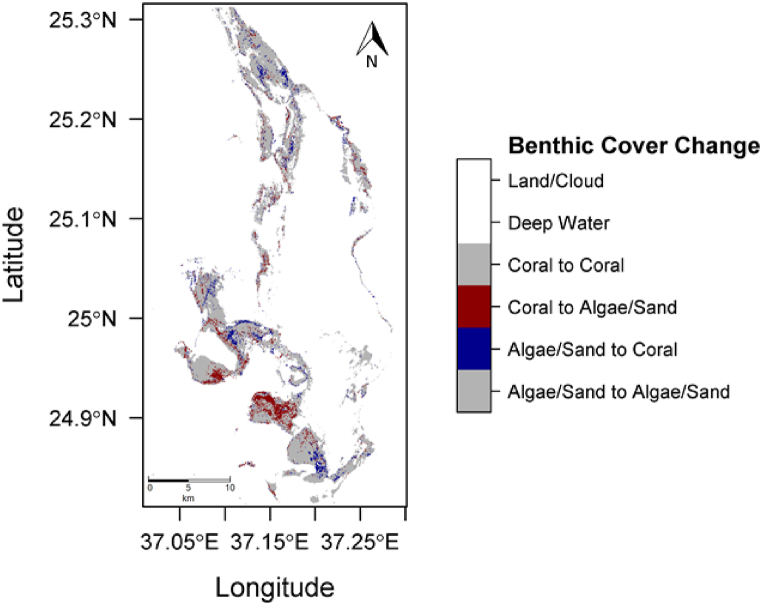
Change detection analysis between 2000 and 2018 of the Umluj AOI.

The initial state image had 53.5 % of shallow water pixels classified as coral, compared to 51.7 % in the final state image. Fig. S3 displays the posterior probability maps for the Umluj location for both 2000 (left) and 2018 (right). Fig. 6 shows the predicted class for each pixel in 2000 (left) and 2018 (right). A per-pixel difference map between the initial and final image classifications is presented in Fig. 7.

#### 3.3.3 Al Wajh location

**Fig. 8:**
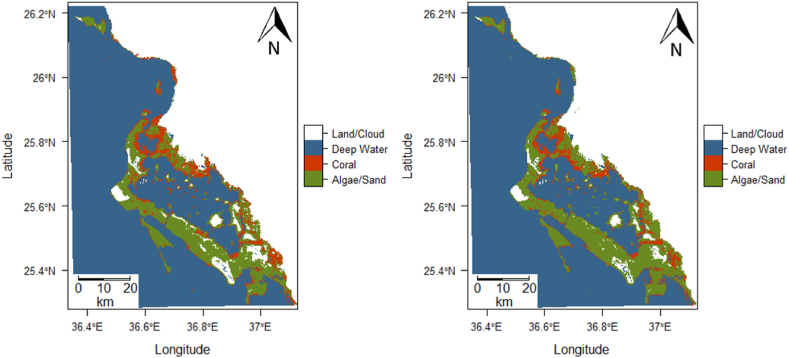
Predicted class membership map for the Al Wajh AOI (left, 2000, and right, 2018).

**Fig. 9:**
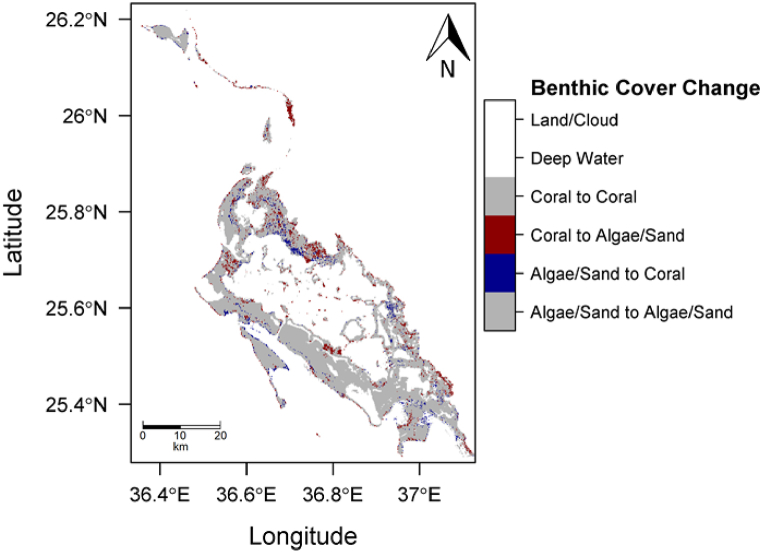
Change detection analysis between 2000 and 2018 of the Al Wajh AOI.

A 13.6 % decrease in coral cover was identified in the Al Wajh location. The Landsat 7 image of Al Wajh from 2000 identified 294,501 pixels (265.05 km^2^) as containing coral. The Landsat 8 image from 2018 revealed 254,567 pixels (229.11 km^2^) as containing coral, representing a 13.6 % decrease in coral cover over the 18-year period. Additionally, 28.2 % of the shallow benthic area in the 2000 image contained coral, compared to 24.4 % in the 2018 image. Fig. S4 displays the posterior probabilities that each pixel contains coral for the initial state (left) and final state (right). The per-pixel classification is shown in Fig. 8 for 2000 (left) and 2018 (right). A per-pixel difference for the Al Wajh location over the 18-year period is displayed in Fig. 9. Table S4 summarizes all the results of the change detection analysis.

## 4 Discussion

This study presents a unique methodology used for building and training a machine learning classifier that can later be generalized to other new locations with data that was never seen by the classifier in the Red Sea area. Compared with the previous our approach in the Pacific Ocean using Landsat 7 and 8 data and the Red Sea using Landsat 5,7, and 8 [[19], [20], [21]], the our new classifier capability was developed using data from one location here, namely the Gulf of Aqaba, followed by evaluating the classifier's performance against the ground truth data from another location, namely, Umluj. This approach reduces site-specific bias by training the model on a dataset more representative of the region's broader ecological characteristics. Instead of memorizing specific geomorphological features, strata, turbidity, and fauna of isolated individual reefs, the robust classifier aims to model a wider range of reef habitats. This results in an analysis that better represents the region as a whole, rather than relying on the localized bias inherent in in-situ investigations. Training the Support Vector Machine (SVM) using regionally representative data, as opposed to site-specific information, enables a more comprehensive analysis of coral reefs and a broad representation of coral habitats, aiding our classifier's robustness and generalizability. This method expands the scope of coral reef studies and enhances the model's applicability across diverse reef environments, with potentially producing a highly consistent evaluation of percent cover, frequency of occurrence, benthic community taxonomic composition, and relative generic richness.

Stuart-Smith (2021) [60] emphasized the importance of a systematic approach to habitat classification, specifically highlighting the role of habitat loss and range shifts in promoting generalization among reef fishes. Roelfsema (2018) and Garza-Pérez (2004) [61,62] provide practical examples of this approach, with Roelfsema [61] demonstrating the integration of ecological coral habitat mapping and empirical modeling, and Garza-Pérez [62] using spatial modeling and remote sensing to predict and classify coral reef habitats. These studies collectively support the idea that a systematic, integrated approach to habitat analysis, as proposed by the authors, can provide a broader scope of coral reef habitat analyses. Based on the above, it is evident that our approach provides a significantly broader scope of coral reefs habitat analyses through the generalization attribute. This makes our approach quite robust and flexible, with an AUC of 0.8432 to scale way beyond limitations posed by the in-situ-based analysis only. Therefore, it is quite clear that the natural evolution of in-situ-based analyses would involve incorporating larger scopes of data spatially and temporally.

Our methodology addresses the challenge of generalizing and extrapolating results from a trained machine learning classifier to a larger extent. To achieve efficient generalization beyond in-situ applications, proper and broader training using an adequate dataset that represents coral habitats throughout a region is essential. The principal challenge of our proposed methodology is that any classifier trained on a diverse set of coral reef data from multiple locations will not achieve the same accuracy as in-situ models. This is because a more robust model is inherently not site-specific and, therefore, cannot be trained on the unique features of an isolated location. This outcome is expected since the model is designed to address various areas rather than the limited scope of a single in-situ investigation. In-situ approaches rely on localized coral features and conditions, resulting in higher accuracy. Furthermore, incorporating site-specific geomorphology and local coral fauna into model training is essential. Consequently, the developed algorithm might underperform when introduced to new data from outside the immediate area of training. However, in-situ classifiers perform exceptionally well when used on data from the specific location they were trained on. In summary, our methodology focuses on developing a generalized machine learning classifier for coral reef habitats across multiple locations, which sacrifices site-specific accuracy for broader applicability and robustness.

## 5 Conclusions

This paper presents a time-based change detection analysis over an 18-year period across three Red Sea locations. Initially, a classifier was trained on data from the Gulf of Aqaba, achieving an accuracy of 78.22 % when compared to ground truth observations. This robust classifier was then applied to the Umluj location to predict coral cover, resulting in 72.73 % of ground truth observations being correctly classified. Subsequently, a strong classifier was developed using ground truth data from both the Gulf of Aqaba and Umluj, and applied to a third site, Al Wajh.

Change detection analysis was conducted for all three locations by comparing Landsat 7 data from 2000 with Landsat 8 data from 2018. The initial state benthic type was determined for each pixel in the 2000 images using the classifier, and the final state benthic type was determined for each pixel in the 2018 images. A per-pixel change detection analysis over the 18-year period was performed. The analysis revealed a reduction in coral reef habitat of 117.20 km^2^ in the Gulf of Aqaba, 3.46 km^2^ in Umluj, and 35.94 km^2^ in Al Wajh. This constitutes a reduction of 11.4 %, 3.4 %, and 13.6 % in the three locations, respectively. Overall, this study demonstrates the effectiveness of using a generalized classifier trained on combined data from multiple locations to detect changes in coral cover over time. The findings underscore the importance of continuous monitoring and robust modeling to understand and mitigate the impacts of environmental changes on coral reef ecosystems.

## Data and code Availability

Landsat data is available for free access through the NASA Landsat Portal https://landsat.gsfc.nasa.gov/data/data-access/. Ground data and code will be made available on request.

## CRediT authorship contribution statement

**Justin J. Gapper:** Writing – review & editing, Writing – original draft, Visualization, Validation, Supervision, Software, Resources, Methodology, Investigation, Formal analysis, Data curation, Conceptualization. **Surendra Maharjan:** Writing – review & editing, Writing – original draft, Resources. **Wenzhao Li:** Writing – review & editing, Writing – original draft, Visualization, Project administration, Methodology, Investigation, Conceptualization. **Erik Linstead:** Writing – review & editing, Visualization, Validation, Methodology, Investigation. **Surya P. Tiwari:** Supervision, Methodology, Investigation. **Mohamed A. Qurban:** Methodology, Investigation, Data curation. **Hesham El-Askary:** Supervision, Methodology, Investigation, Funding acquisition, Conceptualization.

## Declaration of competing interest

The authors declare the following financial interests/personal relationships which may be considered as potential competing interests: Hesham El-Askary reports financial support was provided by US Department of Education. If there are other authors, they declare that they have no known competing financial interests or personal relationships that could have appeared to influence the work reported in this paper.
