# Supplementary material for: A generalized machine learning model for long-term coral reef monitoring in the Red Sea
Source: Heliyon. 2024 Sep 21;10(18):e38249. doi: 10.1016/j.heliyon.2024.e38249 (PMC11458965; doi:10.1016/j.heliyon.2024.e38249)
Supplement: Multimedia component 1 [file mmc1.docx]

**A Generalized Machine Learning Model for Long-Term Coral Reef Monitoring in the Red Sea**

Justin J. Gapper^1^, Surendra Maharjan^1^, Wenzhao Li^1,2^, Erik Linstead^3^, Surya P. Tiwari^4^, Mohamed A. Qurban^5^, Hesham El-Askary^1,2,6^ *

*^1^ Earth Systems Science and Data Solutions Lab, Chapman University, Orange, CA 92866, USA*

*^2^ Schmid College of Science and Technology, Chapman University, Orange, CA 92866, USA*

*^3^ Fowler School of Engineering, Chapman University, Orange, CA 92866, USA*

*^4^ Center for Environment and Water, The Research Institute, King Fahd University of Petroleum and Minerals (KFUPM), Dhahran 31261, Saudi Arabia*

*^5^National Center for Wildlife, Saudi Arabia*

*^6^ Department of Environmental Sciences, Faculty of Science, Alexandria University, Moharem Bek, Alexandria 21522, Egypt*

***** *Corresponding author:* *Hesham El-Askary (*[*elaskary@chapman.edu*](mailto:elaskary@chapman.edu)*)*

Table 1. Summary of Data Used.

| **Site**  **(Figure 1)** | **Path/**  **Row** | **Site** | **Latitude-**  **Longitude** | **Final**  **Scene**  **Date** | **Baseline**  **Scene**  **Date** | **AOI Dimensions** | **Ground Truth**  **Points** |
| --- | --- | --- | --- | --- | --- | --- | --- |
| **1** | 174/041 | Gulf of Aqaba | 27°57′N 34°50′E | 11/7/2018 | 3/18/2000 | 65.7×64.7-km | 1,085 |
| **2** | 172/043 | Umluj | 25°00′N 37°10′E | 12/11/2018 | 2/17/2000 | 55.3×28.4-km | 196 |
| **3** | 172/042 | Al Wajh | 25°35′N 36°48′E | 2/26/2018 | 6/24/2000 | 104.1×77.7-km | - |

Table S2. SVM classifier performance assessment by site and for the consolidated model.

| **Description** | **Gulf of Aqaba** | **Umluj** | **Consolidated Model** |
| --- | --- | --- | --- |
| Accuracy | 78.22% | 72.73% | 70.98% |
| Precision | 0.7664 | 0.7500 | 0.6992 |
| Recall | 0.8119 | 0.6818 | 0.7366 |
| Specificity | 0.7525 | 0.7727 | 0.6830 |
| F-measure | 0.7885 | 0.7143 | 0.7174 |
| Kappa | 0.5644 | 0.4545 | 0.4196 |

Table S3: Confusion Matrix by Site and the Consolidated Model.

|  |  |  | **Ground Truth Labels** | |
| --- | --- | --- | --- | --- |
|  |  |  | **Coral** | **Not Coral** |
| **Predicted Class** |  |  | **Gulf of Aqaba** | |
|  | **Coral** |  | 164 | 50 |
|  | **Not Coral** |  | 38 | 152 |
|  |  |  | **Umluj** | |
|  | **Coral** |  | 15 | 5 |
|  | **Not Coral** |  | 7 | 17 |
|  |  |  | **Consolidated Model** | |
|  | **Coral** |  | 165 | 71 |
|  | **Not Coral** |  | 59 | 153 |

Table S4: Change Detection Analysis by Site.

|  |  |  | **Initial Class**  (pixel count) | |  | **Initial Class**  (${km}^{2}$) | |
| --- | --- | --- | --- | --- | --- | --- | --- |
|  |  |  | **Coral** | **Not Coral** |  | **Coral** | **Not Coral** |
| **Final Class** |  |  | **Gulf of Aqaba** | |  | **Gulf of Aqaba** | |
|  | **Coral** |  | 86,843 | 43,382 |  | 78.16 | 39.04 |
|  | **Not Coral** |  | 60,171 | 91,906 |  | 54.15 | 82.72 |
|  |  |  | **Umluj** | |  | **Umluj** | |
|  | **Coral** |  | 93,299 | 16,144 |  | 83.97 | 14.53 |
|  | **Not Coral** |  | 19,985 | 82,151 |  | 17.99 | 73.94 |
|  |  |  | **Al Wajh** | |  | **Al Wajh** | |
|  | **Coral** |  | 208,448 | 46,119 |  | 187.60 | 41.51 |
|  | **Not Coral** |  | 86,053 | 702,610 |  | 77.45 | 632.35 |

**Figure S1.** SVM Classifier training and change analysis process flow.


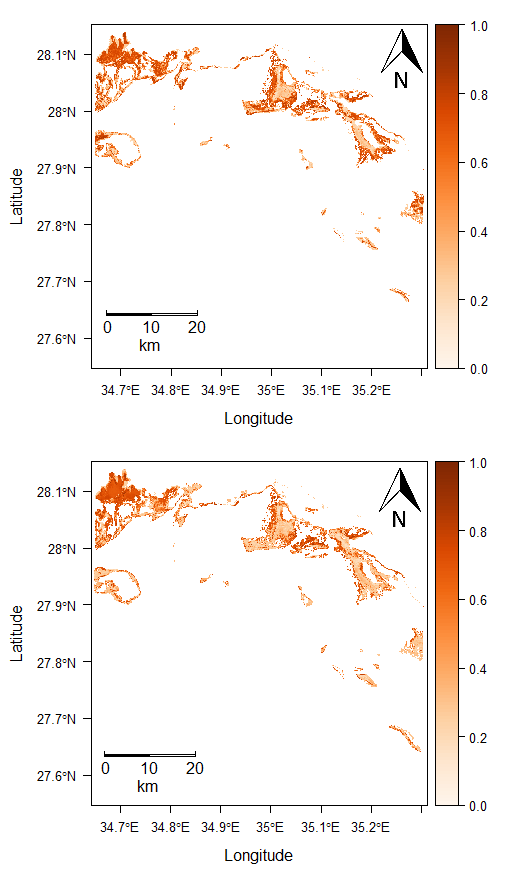


**Figure S2.** Posterior probability map for the Gulf of Aqaba AOI (top, 2000, and bottom, 2018).


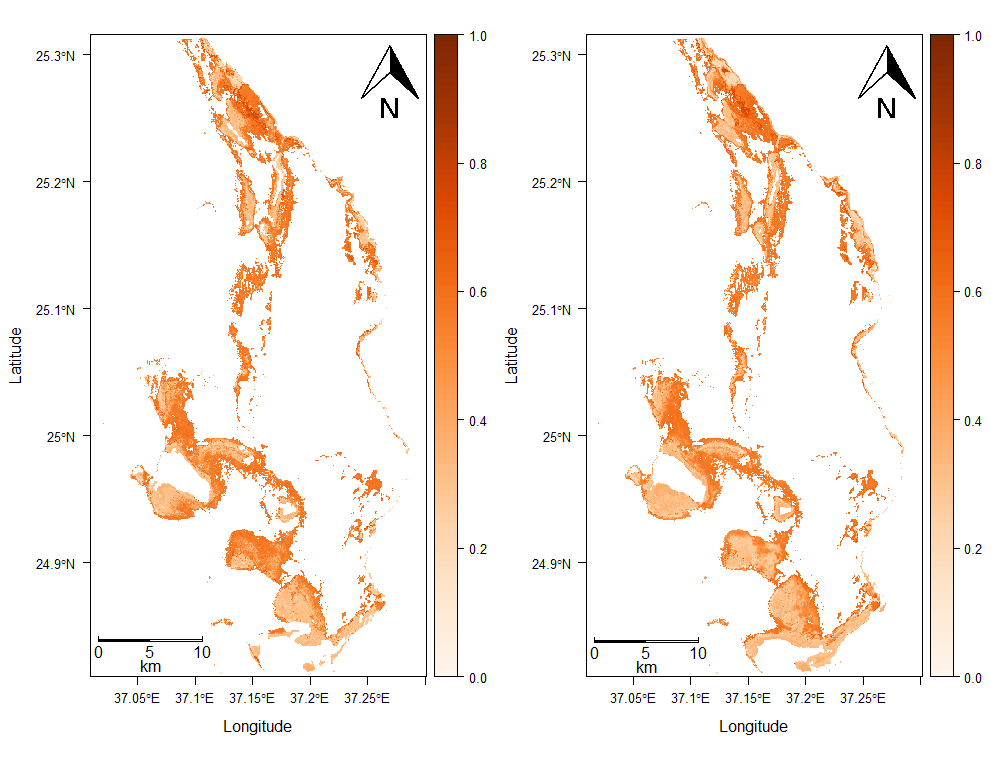


**Figure S3.** Posterior probability map for the Umluj AOI (left, 2000 and right, 2018).


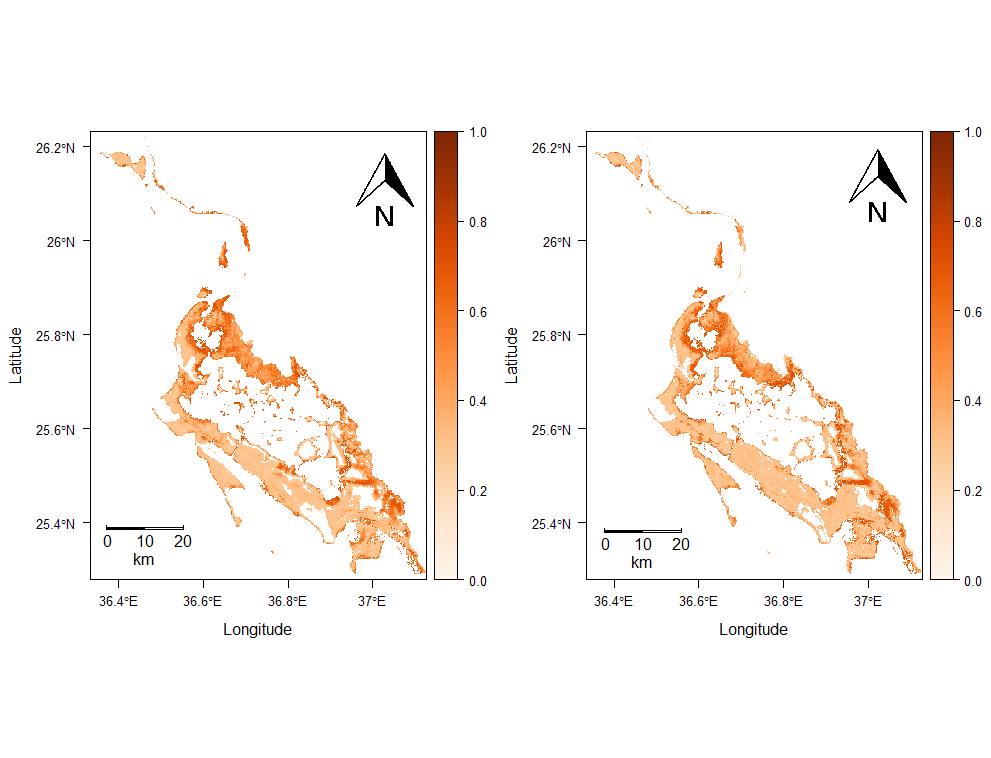


**Figure S4.** Posterior probability map for the Al Wajh AOI (left, 2000, and right, 2018).
